# Analysis of hepatic transcriptome demonstrates altered lipid metabolism following *Lactobacillus johnsonii* BS15 prevention in chickens with subclinical necrotic enteritis

**DOI:** 10.1186/s12944-018-0741-5

**Published:** 2018-04-20

**Authors:** Xiaodan Qing, Dong Zeng, Hesong Wang, Xueqin Ni, Jing Lai, Lei Liu, Abdul Khalique, Kangcheng Pan, Bo Jing

**Affiliations:** 1 Animal Microecology Institute, College of Veterinary, Sichuan Agricultural University, Chengdu, 611130 China; 2 Key Laboratory of Animal Disease and Human Health of Sichuan Province, Chengdu, Sichuan China

**Keywords:** *Lactobacillus johnsonii*, Subclinical necrotic enteritis, Hepatic transcriptome, Lipid metabolism pathways

## Abstract

**Background:**

Subclinical necrotic enteritis (SNE) widely outbreaks in chickens which inflicted growth-slowing, causing enormous social and economic burdens. To better understand the molecular underpinnings of SNE on lipid metabolism and explore novel preventative strategies against SNE, we studied the regulatory mechanism of a potential probiotic, *Lactobacillus johnsonii* BS15 on the lipid metabolism pathways involved in chickens with SNE.

**Methods:**

One hundred eighty one-day-old chickens were randomly divided into three groups and arranged with basal diet (control and SNE group). Added with BS15 (1 × 10^6^ cfu/g) or Man Rogosa Sharpe (MRS) liquid medium for 28 days. The hepatic gene expression of each group was then measured using high-throughput analysis methods (RNA-Seq). Quantitative real-time PCR (qRT-PCR) was used to detect the expression changes of the related genes.

**Results:**

The results showed that there are eleven lipid metabolic pathways were found during the prevention of BS15 treatment in SNE chickens by RNA-Seq, including the peroxisome proliferator-activated receptor (PPAR) signaling pathway and arachidonic acid metabolism. BS15 notably facilitated the expressions of fatty acid binding protein 2 (FABP2), acyl-CoA synthetase *bubblegum* family member 1 (ACSBG1), perilipin 1 (PLIN1) and perilipin 2 (PLIN2), which were involved in PPAR signaling pathway of SNE chickens. Besides, suppression of phospholipase A2 group IVA (PLA2G4A) in arachidonic acid metabolism was observed in SNE chickens after BS15 prevention. The expression patterns of FABP2, ACSBG1, PLIN1, PLIN2 and PLA24G in qRT-PCR validation were consistent with RNA-Seq results.

**Conclusions:**

These findings indicate that SNE may affect the hepatic lipid metabolism of chickens. Meanwhile, BS15 pretreatment may provide a prospective natural prophylaxis strategy against SNE through improving the PPAR signaling pathway and arachidonic acid metabolism.

**Electronic supplementary material:**

The online version of this article (10.1186/s12944-018-0741-5) contains supplementary material, which is available to authorized users.

## Background

Subclinical necrotic enteritis (SNE) of broiler chickens is characterized by the intestinal damage and growth-slowing without mortality [1]. The residues of enterotoxigenic *Clostridium perfringens* that is the most predominant causes of SNE menaces the public health via the food chain [2, 3]. As a result of the ban of subtherapeutic antibiotics usage in the European Union, the incidence of SNE has further increased in recent years [4], which made numerous social economic losses via chronic impairing the lipid metabolism of chicken [5] and decreasing the quality of chicken meat [6]. Accordingly, finding valid alternatives to antibiotics has gained in importance [7, 8]. To date, as one of the best ideal alternatives, probiotics exhibit positive influences on the growth development and necrotic enteritis diseases in broiler chickens [9–12], which are proposed as an attractive option for treating SNE. Based on this, we firstly discovered that *Lactobacillus johnsonii* BS15 (CCTCC M2013663) strain exhibited the beneficial effects on lipid metabolism in the previous studies, resulting in preventing non-alcoholic fatty liver disease in obese mice [13]. Then we applied this strain to chickens and found similar conditions, including it improved the meat nutritional value through altering the fatty acid composition [14], and promoted growth performance and lowered fat deposition in broilers [15]. Subsequently, we demonstrated that the aberrations of lipid metabolism during subclinical *Clostridium perfringens* infection was obviously ameliorated after BS15 prevention through controlling the lipid deposits and fatty acid composition [6].

Based on the rationale mentioned above, we speculated that *L. johnsonii* BS15 may prevent SNE associated with regulating the hepatic lipid metabolism. However, information is still limited on the molecular underpinnings of SNE pathogenesis in hepatic transcriptome, and how BS15 exerts its beneficial effects on hepatic lipid metabolism of SNE chickens has not been reported. To verify this speculation, we have indicated that BS15 prevention really improved the hepatic lipid metabolism of chickens with SNE [5], nevertheless, its molecular mechanism remains unrevealed. Therefore, RNA sequencing was undertaken to characterize the hepatic transcriptome in the present study for better understanding the molecular mechanism about lipid metabolism in SNE chickens and providing a novel preventative strategy against SNE.

## Results

### Preventive effect of BS15 on the SNE infection chicken model

**Fig. 1:**
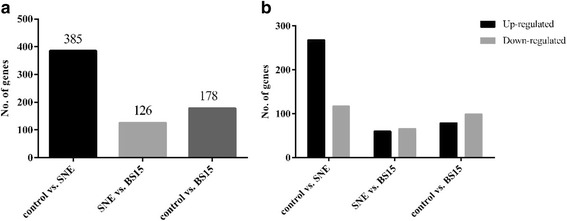
RNA-seq results among control, subclinical necrotic enteritis (SNE) and Lactobacillus johnsonii BS15 (BS15) groups. **a**, screened genes among control, SNE and BS15. 385 differentially expressed genes were screened in control vs. SNE groups, 126 differentially expressed genes were screened in SNE vs. BS15 groups, 178 differentially expressed genes were screened in control vs. BS15. **b**, up-regulated and down-regulated genes in hepatic transcriptome. 268 genes were up-regulated from control to SNE, 60 genes were up-regulated from SNE to BS15; while 117 genes were down-regulated from control to SNE, 66 genes were down-regulated from SNE to BS15

To explore the molecular preventive mechanism of BS15, we carried out a hepatic gene expression study with a SNE infection chicken model. There is no death records during the whole experiment, and the some rudimentary parameters results of growth performance and serum were described in the previous report [5]. Besides, the screened genes among control, SNE and BS15 was showed in Fig. 1.

### Genes and pathways related to lipid metabolism associated with SNE disease

**Fig. 2:**
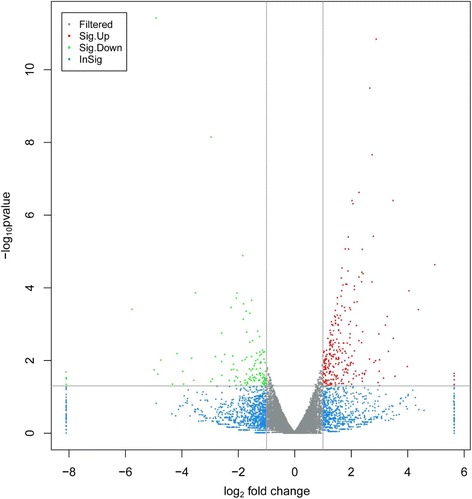
Volcano plot of control group vs. subclinical necrotic enteritis group. The 385 differentially expressed genes that fall above our threshold value are pictured in red (Up-regulated) and green (Down-regulated). The blue dots are the insignificantly different expressed genes

We analyzed alterations in lipid metabolism gene expression in three SNE and three normal chicken liver tissue specimens using the powerful RNA sequencing technology. 385 genes as being differentially expressed in the SNE liver samples as compared with control animals’ (*p* < 0.05) were observed (Fig. 2, Additional file 1: Table S1). Gene ontology (GO) functional analysis was used to decipher the major biological processes affected among the differentially expressed genes. From this analysis, we found that SNE infection altered a multitude of biological processes relating to the metabolic regulation of lipid, including fatty acid, cholesterol, lipid storage, phosphatidylinositol, steroid, phospholipid and triglyceride (*p* < 0.05) (Additional file 2: Table S2). In addition, some biological processes were also found to be dysregulated and included regulation of cellular response to oxidative stress, calcium ion transmembrane transport, inflammatory response, and so on, manifesting that there exists inflammation of the liver, which was responsible for successful SNE infection model. More importantly, the kyoto encyclopedia of genes and genomes (KEGG) database revealed ten genes that were enriched in the hepatic transcriptome that participated in 12 pathways related to lipid metabolism, including steroid biosynthesis, insulin signaling pathway, adipocytokine signaling pathway, PPAR signaling pathway, and so on (Table 1).

**Table 1:** Important regulatory genes and pathways related to lipid metabolism in comparison of control group and SNE group

Pathway	*P*-value	Pathway ID	Genes
Steroid hormone biosynthesis	0.05007	gga00140	CYP17A1; CYP1B1
Steroid biosynthesis	0.07212	gga00100	LIPA
Ether lipid metabolism	0.08339	gga00565	LPCAT1; PLA2G4A
alpha-Linolenic acid metabolism	0.10632	gga00592	PLA2G4A
Linoleic acid metabolism	0.13425	gga00591	PLA2G4A
beta-Alanine metabolism	0.16359	gga00410	ALDH1A3
Arachidonic acid metabolism	0.34997	gga00590	PLA2G4A
Glycerophospholipid metabolism	0.41918	gga00564	LPCAT1; PLA2G4A
PPAR signaling pathway	0.49544	gga03320	PLIN2
Insulin resistance	0.50696	gga04931	PIK3R5; PRKCB
Adipocytokine signaling pathway	0.52206	gga04920	TNFRSF1B
Insulin signaling pathway	0.84921	gga04910	PIK3R5

### Genes and pathways mediating the preventive effects of BS15 treatment

**Fig. 3:**
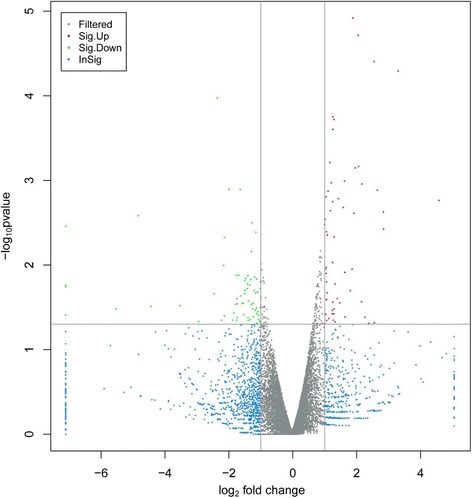
Volcano plot of subclinical necrotic enteritis group vs. Lactobacillus johnsonii BS15 group. The 126 differentially expressed genes that fall above our threshold value are pictured in red (Upregulated) and green (Down-regulated). The blue dots are the insignificantly different expressed genes

In order to identify the certain genes and pathways associated with BS15 prevention, KEGG analysis of pathway enrichment was conducted with the differentially expressed genes between the SNE infection and BS15 treatment groups. BS15 prevention created far-reaching influence on genes expression in the livers of SNE chickens. Compared with the SNE group, significantly elevated expression of 60 genes and obviously decreased expression of 66 genes were found in the livers of BS15-disposed chickens (Fig. 3. Additional file 3: Table S3). What’s more, the results of enriched pathways analysis highlighted the changes related to lipid metabolism, including glycerophospholipid metabolism, adipocytokine signaling pathway, glycerolipid metabolism, fatty acid metabolism, PPAR signaling pathway, and so on (Table 2).

**Table 2:** Genes and pathways relevant to lipid metabolism mediating the preventive effects of BS15 treatment bases on SNE chickens

Pathway	*P*-value	Pathway ID	Genes
PPAR signaling pathway	0.00107	gga03320	ACSBG1; PLIN1; PLIN2
Fatty acid biosynthesis	0.00443	gga00061	ACSBG1
Arachidonic acid metabolism	0.00472	gga00590	PLA2G4A; PTGES2
alpha-Linolenic acid metabolism	0.01043	gga00592	PLA2G4A
Linoleic acid metabolism	0.01368	gga00591	PLA2G4A
Fatty acid degradation	0.02567	gga00071	ACSBG1
Ether lipid metabolism	0.03364	gga00565	PLA2G4A
Fatty acid metabolism	0.04244	gga01212	ACSBG1
Glycerolipid metabolism	0.06227	gga00561	LIPC
Adipocytokine signaling pathway	0.08463	gga04920	ACSBG1
Glycerophospholipid metabolism	0.14045	gga00564	PLA2G4A

We also compared the control group with the BS15 group, and found that there were 15 lipid metabolic pathways enriched (Table 3). According to the literature and the above data analysis, We found phospholipase A2 group IVA (PLA2G4A) was persistently significant differentially expressed in two comparisons. Besides, we also found some other genes differential expression relevant to lipid metabolism in comparison of control versus SNE groups or SNE versus BS15 groups, such as fatty acid binding protein 2 (FABP2), acyl-CoA synthetase *bubblegum* family member 1 (ACSBG1), perilipin 1 (PLIN1), perilipin 2 (PLIN2), and so on.

**Table 3:** Important regulatory genes and pathways related to lipid metabolism in comparison of control group and BS15 group

Pathway	*P*-value	Pathway ID	Genes
Steroid biosynthesis	7.64E-09	gga00100	CYP51A1; DHCR7; LIPA; MSMO1; SQLE
Fatty acid metabolism	0.00093	gga01212	ACSBG1; HADHA; SCD5
Fatty acid elongation	0.00113	gga00062	ELOVL1; HADHA
Biosynthesis of unsaturated fatty acids	0.00131	gga01040	HADHA; SCD5
PPAR signaling pathway	0.00332	gga03320	ACSBG1; FABP2; SCD5
Steroid hormone biosynthesis	0.00374	gga00140	CYP17A1; CYP1B1
Fatty acid degradation	0.00450	gga00071	ACSBG1; HADHA
Fatty acid biosynthesis	0.00801	gga00061	ACSBG1
Adipocytokine signaling pathway	0.02749	gga04920	ACSBG1; SOCS3
Butanoate metabolism	0.02841	gga00650	HADHA
beta-Alanine metabolism	0.03058	gga00410	HADHA
Ether lipid metabolism	0.05823	gga00565	LPCAT1
Glycerophospholipid metabolism	0.22438	gga00564	LPCAT1
Insulin resistance	0.27196	gga04931	SOCS3
Insulin signaling pathway	0.35875	gga04910	SOCS3

### Validation of next-generation sequencing data using qRT-PCR

**Fig. 4:**
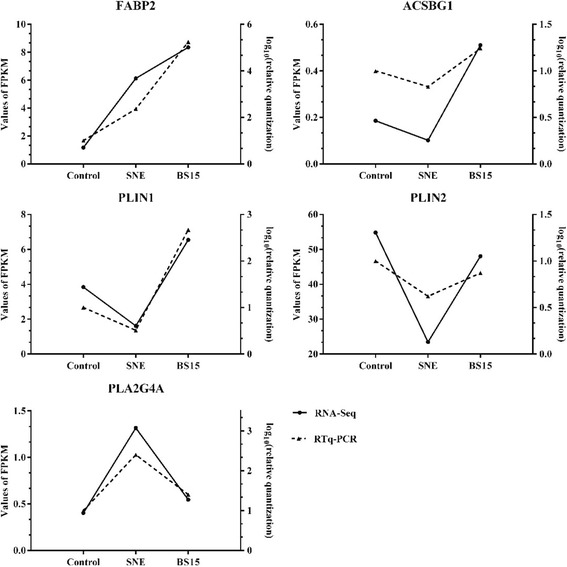
Expression changes of FABP2, ACSBG1, PLIN1, PLIN2 and PLA2G4A. The expression levels of FABP2, ACSBG1, PLIN1, PLIN2 and PLA2G4A during chicken liver from RNA-Seq results are same with the expression trends of these genes from quantitative real-time (qRT)-PCR results. FPKM, fragments per kb per million reads

To validate the RNA sequencing results, qRT-PCR analysis was used to determine the expression patterns of some target genes that were normalized by the expression of glyceraldehyde-3-phosphate dehydrogenase (GAPDH). These genes consisted of overexpressed and underexpressed genes (FABP2, ACSBG1, PLIN1, PLIN2, PLA2G4A) in SNE or BS15 groups. The results showed in Fig. 4, confirming these genes are crucial in regulation of lipid metabolism in SNE infection chickens.

## Discussion

Subclinical necrotic enteritis, a enteric bacterial disease, is known to widely hamper the growth of chicken, contaminate chicken meat, and cause huge economic losses, suggesting that not only the poultry but also the public health is exposed to this harmful subclinical disease. In previous study, we have demonstrated that SNE infection could cause the deregulation of lipid metabolism in chicken liver, and *L. johnsonii* BS15 pretreatment can alleviate this damage [5]. Then, we hypothesized that BS15 may prevent SNE by ameliorating hepatic lipid metabolism. To test this hypothesis, we used chickens as models to excavate the key functional genes and pathways related to hepatic lipid metabolism of SNE chickens following BS15 supplementation in daily diet. Our results were consistent with this assumption, and more importantly, we found that adding BS15 in the whole experiment could improve the lipid metabolism, mainly by regulation of the peroxisome proliferator-activated receptor (PPAR) signaling pathway and arachidonic acid metabolism.

The rudimentary parameters values of growth status, such as feed conversion rate and the abdominal rate, as well as some concentrations of serum biochemical indicators (alanine aminotransferase, aspartate transaminase, total cholesterol, high-density lipoprotein cholesterol), were significantly increased in the SNE chickens [5], manifesting that the success of SNE experimental model which is indispensable for the follow-up trial. Meanwhile, chicken characteristics have showed that SNE infection could indeed bring adverse effects on the lipid metabolism of chickens. Similar phenomenon was also observed by Wang et al. (2017) [6] that demonstrated subclinical *C. perfringens* infection elevated fatty acid and lipid production, resulting in lipid accumulation in the muscle. However, there was a detente after BS15 preconditioning, which caught our attention. The liver of chickens is a vital organ where the majority of lipid metabolism process occurs, and considerable evidence support that the interrelationships between liver and some bowel diseases. For example, ascites can lead broiler liver oxidation damage and energy generation obstruction [16]. Also, Coble et al. (2013) [17] found that *Salmonella* enteritidis infection spawned many pronounced response on inflammation, metabolic pathways, and mitochondria-mediated apoptosis in broiler liver. Based on the above multi-factors, we focused on the lipid metabolism in hepatic transcriptome of SNE chickens prevented by BS15.

Numerous researches showed that the activation of PPAR signaling pathway at the transcriptomic level is closely associated with fatty acids biosynthesis and metabolism [18], and PPAR signaling pathway also touches on ameliorating insulin sensitivity [19], maintaining energy balance [20], regulating the fatty acid oxidation and ketogenesis [21]. In addition, adipocyte differentiation is adjusted by miscellaneous transcription factors, notably PPARs [22], whose expressions are susceptible due to altering different diets in broiler livers [10, 23]. In present study, our data showed that BS15 treatment boosted the gene expression levels of enzymes responsible for oxidation and transportation of fatty acid in the PPAR signaling pathway, including FABP2, ACSBG1, PLIN1 and PLIN2.

FABP2 predominantly expressed in enterocytes belongs to FABPs, which is cytoplasmic proteins touched upon intracellular fatty acid transport and metabolism [24, 25]. Previous studies have identified that FABP2 plays a key role in the absorption and intracellular transport of dietary long-chain fatty acids [26, 27], and the explanation of observed fatty acids alterations in Major Depressive Disorder [28]. Further, FABP2 Ala54Thr polymorphism, a mutant phenotype, is closely related to insulin resistance and abnormal lipid metabolism, which are the main risk factors for cardiovascular disease [29]. These observations were consistent with our findings that the expression of FABP2 was statistically significantly up-regulated (Fold Chang, FC: 6.372) by BS15 compared with the control group, although there was no apparent response in the SNE chicken group. This phenomenon presumably results from the indubitable facilitation of BS15 on hepatic fatty acid transport and metabolism in SNE chickens.

Before participating in most catabolic and anabolic reactions, free fatty acids must be activated to their CoA thioesters [30, 31]. Processes such as incorporation of fatty acids into fatty acid unsaturation or elongation, degradation of fatty acids by oxidation, which all need to activate the fatty acid substrates. Most activations are catalyzed by acyl-CoA synthetases that play a virtual role in fatty acid and lipid metabolism in animals [32, 33]. Further analysis in this study, we found ACSBG1 is another key gene which persistently differentially expressed in three groups. ACSBG1 was proved to be the one has robust acyl-CoA synthetase activity and capable of activating both long- and very long-chain fatty acid substrates [34]. Fruit flies lacking this gene have elevated tissue levels of saturated very long-chain fatty acids [35], more importantly, ACSBG1 characterized by regulation of nerve impulses [36]. In current study, ACSBG1 overexpressed (FC: 2.506) in the BS15 chickens compared the SNE group, involving in PPAR signaling pathway enrichment relevant to lipid metabolism, suggesting the acceleration of fatty acid transport in SNE chickens liver controlled by BS15.

However, as the analysis of transcriptome goes on, we found two other key genes, PLIN1 and PLIN2, which may have an important regulatory effect on PPAR signaling pathway. PLIN1 and PLIN2 are both the members of PAT protein family which is a key protein that regulates the formation of lipid droplet [37, 38]. Previous researches demonstrated that phosphorylated PLIN1 activated lipolytic activity and hydrolysis of triacylglycerides [39, 40]. Relatively lower expression of PLIN2 can promote the reduction of hepatic fibrosis and enhance insulin sensitivity, while simultaneously suppressing PLIN2 and PLIN3 can lead to insulin resistance [41]. Whence the role of the PAT family protein in the disease associated with lipid droplet and dyslipidemia is still not really clearly in the current researches. Our sequencing data showed that the up-regulated of PLIN1 (FC: 0.267) and PLIN2 (FC: 0.493) were observed in BS15 chickens group compared to the SNE group, and there were no significant differences between control and BS15 groups in terms of these two genes comparison. To some extent, these results suggest that *L. johnsonii* BS15 addition may slow down the fat cracking to recuperate the metabolic rate and body resistance, inasmuch as the growth of SNE chickens was generally poor [5, 6]. However, this feature of BS15 is far from sufficient to cover the characteristic of BS15 facilitating the transfer of fatty acids in statistics.

On the other hand, the membranes of cells and suborganelles are rich in easily oxidized phospholipids that is the initiation site of fatty acid oxidation. Phospholipase A2 (PLA2) with special selectivity of acyl are the key rate-limiting enzymes of phospholipids oxidation [42]. Besides, PLA2 can catalyze the hydrolysis of membrane phospholipid glycerin to produce free fatty acids and lysophospholipid, such as arachidonic acid and linoleic acid [43]. As a n-6 highly unsaturated fatty acid, arachidonic acid is an important inflammatory lipid media and can participate in the systemic stress responses and inflammation [44, 45], activate PPARγ with its metabolites [46], affect the transcription of lipid metabolism related genes and regulate the synthesis and storage of fatty acids [47]. PLA2G4A is the main isotype of the release of arachidonic acid. Our results manifested that the significantly overexpression of PLA2G4A (FC: 2.989) in SNE chickens liver compared to the control group, suggesting that SNE infection accelerated the accumulation of arachidonic acid, which may cause large amount of fatty acid oxidation and the formation of lipid peroxides, aggravating liver cell injury. This situation was consistent with the previous researches that indicated that SNE may induce the inflammation in liver [3]. However, an obviously suppression of PLA2G4A (FC: 2.462) in SNE chickens under the BS15 pretreatment was observed, and there was no significantly differential between BS15 and control groups, implying BS15 supplementation can certainly alleviate inflammation through regulating the arachidonic acid metabolism of SNE chickens liver.

## Methods

### Insolation and cultivation of strains

The insolation of *Lactobacillus johnsonii* BS15 (CCTCC M2013663) strain was carried out from homemade yogurt collected from the Hongyuan Prairie, Aba Autonomous Prefecture, China. And the quantities of BS15 were assessed by heterotrophic plate counts after cultivating in MRS liquid medium at 37 °C for 36 h in the anaerobic cabinet. Afterwards, the probiotic cells were collected, washed with saline, and suspended in phosphate buffered saline (PBS, pH 7.0) for further trial use. The premium additive concentration of BS15 in diet was maintained at a level of 1 × 10^6^ colony-forming unit (cfu) per gram to ensure the availability of cells throughout the experimental period.

A *Clostridium perfringens* (CVCC2030) strain, originated from the intestine of a chicken with severe necrotic enteritis, was obtained from China Veterinary Culture Collection Center and characterized as a NetB toxin positive type A strain. The bacteria were cultivated in cooked meat medium at 37 °C for 24 h under anaerobic environment, and then the strain was aseptically stored in fluid thioglycollate broth overnight at the same environment before undergoing the inoculation of chicken.

### Animal maintenance and subclinical necrotic enteritis trials

Cobb 500 male chickens were purchased from the Chia Tai broiler hatchery (Chengdu, China). The protocols for animals studies were reviewed and approved by the Institutional Animal Care and Use Committee of Sichuan Agricultural University. Chickens were maintained, with 24 h a day light, under 24 °C after gradually decreased by 3 °C per a week from 33 °C. Chickens (one-day-old) were randomized into three groups (six replicates per group, ten individuals per replicate) which are as follows: normal control group (NC), SNE group (SNE, SNE experiment model), BS15 preventative group (BS15, *L. johnsonii* BS15 at a dose of 10^6^ cfu per gram). All groups of chickens were allowed drinking water and feed ad libitum unless otherwise stated. The feed was based on the NRC (1994) and shown in Table 4.

**Table 4:** Composition of the basal diets for broilers

Ingredient^a^	diet (%)
Ground yellow corn	56.0
Soybean meal	37.0
Soybean oil	3.66
Ground limestone	0.57
Dicalcium phosphate	1.80
Salt	0.30
Choline chloride	0.10
DL-Met	0.24
Micronutrients^b^	0.33
Calculated nutrients level (%)	
ME (MJ kg-1)	12.39
CP	21.17
Lys	1.19
Met	0.50
Met + Cys	0.86
Ca	0.85
Nonphytate P	0.44

^a^Ingredient and nutrient composition are reported on as-fed basis

^b^Micronutrients are provided per kilogram of diet: vitamin A (all-trans retinol acetate), 12,500 IU; cholecalciferol, 2500 IU; vitamin E (all-rac-a-tocopherol acetate), 18.75 IU; vitamin K (menadione Na bisulfate), 5.0 mg; thiamin (thiamin mononitrate), 2.5 mg; riboflavin, 7.5 mg; vitamin B6, 5.0 mg; vitamin B12, 0.0025 mg; pantothenate, 15 mg; niacin, 50 mg; folic acid, 1.25 mg; biotin, 0.12 mg; Cu (CuSO4·5H2O), 10 mg; Mn (MnSO4·H2O), 100 mg; Zn (ZnSO4·7H2O), 100 mg; Fe (FeSO4·7H2O), 100 mg; I (KI), 0.4 mg; Se (Na2SeO3), 0.2 mg

All chickens were fed on normal diet for first seven days to stabilize their metabolic condition. From day 8 onwards, *L. johnsonii* BS15 bacteria were mixed in the feed of the BS15 preventative group at a concentration of 10^6^ cfu per gram. The dose of 20,000 *Eimeria acervulina* oocysts and 5000 *Eimeria maxima* oocysts (Guangdong Academy of Agricultural Sciences, Guangzhou, China) was given in chickens by gastric infusion as the preliminary infection of SNE on day 15, while the NC group chickens received the same amount of sterile PBS instead. From day 18 to 22, all *Eimeria* oocysts infected chickens were orally challenged (two times a day) with 1 ml of a fresh medium culture containing 2.2 × 10^8^ cfu *C. perfringens* per milliliter, meanwhile, the unchallenged chickens received sterile fluid thioglycollate medium instead. On day 28 all chickens were euthanized for tissue collection under the the institutional animal care guidelines.

RNA library construction and illumina sequencing.

Total RNA was extracted from chicken livers using RNAiso Plus reagent (TaKaRa, Dalian, China). Fifty nanogram of RNA samples with an RNA integrity number (RIN) greater than seven were used for library construction (Agilent, CA, USA). Nine libraries (three each for the control, SNE treatment and BS15 preventative groups) were established using the TruSeq Stranded mRNA LT Sample Prep Kit in the light of the specifications (Illumina, San Diego, USA). And then paired-end reads sequencing was performed on the HiSeq X Ten system (Illumina, Inc., USA) by Shanghai OE Biotech. Co., Ltd. (Shanghai, China). The obtained results were compared with the database and annotations of every gene for subsequent experimental analysis.

Quantitative real time polymerase chain reaction (qRT-PCR) verification.

The total extracted RNA of chicken livers was then reverse transcribed into cDNA immediately using a Prime ScriptTM RT reagent kit (TaKaRa, Dalian, China) to serve as a template for qRT-PCR verification. qRT-PCR was implemented to determine the expression of differentially target genes according to the the fluorescence quantitative PCR kit instructions, using a CFX96 Real-Time system (Bio-Rad, Hercules, CA, USA) with SYBR® Premix Ex Taq™ II (TaKaRa, Dalian, China). The primer sequences for qRT-PCR verification are listed in Table 5. Fold change relative to normal control group was evaluated by using the 2^-ΔΔCt^ method in Microsoft Excel software.

**Table 5:** Primer sequences used in quantitative qRT-PCR analysis

Gene name	Primer sequence (5 → 3)	Size (bp)
FABP2	F: ATACAGGTGAGTTGAACAGTCGCTT R: TGAAGATAAGTGAGGCTGATTGGT	127
ACSBG1	F: CGAATCAGTGCTGTGTGCTT R: GGCTGAGCGGAAGATAACTG	171
PLIN1	F: TGCTGCTTGTTGAAGAACCACT R:AGGCATTCTGTGATGATTATGTGGT	109
PLIN2	F: GTTGCCAATGCTAAGGGTGT R: ACCACACGACTTCCCAAGAC	194
PLA2G4A	F: ACTTGACCACTTCCCGTGAC R: GGGTTGTGACTGACCGAGTT	250
GADPH	F:GGTGAAAGTCGGAGTCAACGG R:CGATGAAGGGATCATTGATGGC	108

### Statistical analysis

The differentially expressed genes between different samples were compared by the method of reads per kb fragments per million reads (FRKM). False Discovery Rate (FDR) was set at 0.0001 to determine the threshold of the *p*-value in multiple tests. An absolute log_2_ fold-change between conditions ≥0.5 was used as the threshold for significance of the gene expression difference. qRT-PCR data were analyzed by one-way analysis of variance (ANOVA), and multiple comparisons were tested using Duncan’s multiple-range test. A *p*-value of < 0.05 was considered statistically significant differences, and all statistical analyses were performed using SigmaPlot for Social Sciences version 13.

## Conclusion

In conclusion, the data presented here highlights the preventative mechanism of *Lactobacillus johnsonii* BS15 that may contribute to lipid metabolism, mainly by regulation of the PPAR signaling pathway and the arachidonic acid metabolism. And this lipid-targeting BS15 precaution may be a good match for the multi-hit driven SNE pathogenesis. We hope that this study lays the foundation for the innovation of natural products for treating SNE.

### Data availability

The raw counts data obtained in this study were deposited in the NCBI Sequence Read Archive (SRA) under the accession number SRP118769.

## Additional files

Additional file 1: **Table S1.** Details for regulatory genes and pathways related to lipid metabolism in comparison of control group and SNE group. (XLSX 120 kb)

Additional file 2: **Table S2.** Details for genes and pathways relevant to lipid metabolism mediating the preventive effects of BS15 treatment bases on SNE chickens. (XLSX 13 kb)

Additional file 3: **Table S3.** Details for regulatory genes and pathways related to lipid metabolism in comparison of control group and BS15 group. (XLSX 42 kb)

## Abbreviations

ACSBG1: acyl-CoA synthetase *bubblegum* family member 1
FABP2: fatty acid binding protein 2
FC: Fold Chang
FDR: False Discovery Rate
GAPDH: glyceraldehyde-3-phosphate dehydrogenase
KEGG: kyoto encyclopedia of genes and genomes
PLA2G4A: phospholipase A2 group IVA
PLIN1: perilipin 1
PLIN2: perilipin 2
PPAR: proliferator-activated receptor
qRT-PCR: Quantitative real-time polymerase chain reaction
RIN: RNA integrity number
SNE: Subclinical necrotic enteritis
